# Psoas Compartment Block Versus Medial Branch Block for Low Back Pain: A Narrative Review of Anatomical Targets, Clinical Evidence, and Patient Selection

**DOI:** 10.3390/bioengineering13091071

**Published:** 2026-09-15

**Authors:** Chul Hee Jung, Seok Yeon Choi, Dong Ha Lee

**Affiliations:** 1 Department of Orthopaedic Surgery, Aerospace Medical Center, Republic of Korea Air Force, Cheongju 28187, Republic of Korea; 2 Department of Rehabilitation Medicine, Aerospace Medical Center, Republic of Korea Air Force, Cheongju 28187, Republic of Korea; 3 Department of Orthopaedic Surgery, Seoul National University Hospital, 101 Daehakro, Jongno-gu, Seoul 03080, Republic of Korea

**Keywords:** low back pain, medial branch block, psoas compartment block, lumbar plexus block, zygapophysial joint, regional anaesthesia, interventional pain management, patient selection

## Abstract

Background: Low back pain (LBP) remains the leading global cause of years lived with disability, and image-guided nerve blocks occupy a central position in its diagnostic and therapeutic pathway. Two paraspinal targets separated by only a few centimetres of tissue address entirely different neural territories: the medial branch of the lumbar dorsal ramus, addressed by the medial branch block (MBB), and the ventral rami of L1–L4 that form the lumbar plexus within psoas major, addressed by the psoas compartment block (PCB). Despite this anatomical proximity, the two techniques have matured within largely separate literatures—interventional pain medicine for the MBB and regional anaesthesia for the PCB—and have never been compared head-to-head in a randomised trial. Methods: This narrative review searched PubMed/MEDLINE, Embase, Scopus, the Cochrane Library, and Google Scholar to 1 August 2026 and synthesises evidence on the anatomical rationale, technical performance, effectiveness, and safety of both blocks as they apply to patients presenting with LBP. Results: The MBB is supported by extensive diagnostic-accuracy data and guideline endorsement for facet-mediated axial pain, but requires controlled blocks to constrain false-positive rates and confers only short-lived therapeutic benefit. The PCB reliably produces dense lumbar plexus analgesia and has a well-defined perioperative role, yet direct evidence in chronic LBP is confined to small series, and its complication profile—including epidural spread and retroperitoneal haematoma—is materially more demanding. Conclusions: We propose a phenotype-driven selection framework in which the MBB serves posterior-element axial pain while the PCB is reserved for plexus-mediated, hip–spine overlap, and perioperative indications, and we outline the comparative studies required to test it.

## 1. Introduction

Low back pain is the single largest contributor to years lived with disability worldwide. The Global Burden of Disease Study 2021 estimated 619.00 million prevalent cases in 2020 (95% uncertainty interval [UI], 554.00–694.00 million), with a projected rise to 843.00 million by 2050 (95% UI, 759.00–933.00 million) driven principally by population growth and ageing [1]. The burden is concentrated in the working-age population, where it translates directly into lost productivity and early withdrawal from employment [2]. Occupational cohorts subject to sustained axial loading—including military aircrew, transport workers, and manual labourers—are disproportionately represented, and the clinical demand for durable, day-case interventions in these groups is correspondingly high.

The therapeutic difficulty of low back pain (LBP) stems less from a shortage of interventions than from the difficulty of matching an intervention to a pain generator. The lumbar spine contains multiple structures capable of producing overlapping symptom patterns, and no physical examination manoeuvre or imaging finding reliably isolates any one of them. Diagnostic local anaesthetic blockade therefore occupies an unusual position in spinal practice: it is simultaneously a treatment and the principal means of confirming a diagnosis [3,4]. Among the available targets, the zygapophysial (facet) joint has attracted the largest body of evidence, and blockade of its innervating medial branches—the medial branch block (MBB)—has become the reference procedure for identifying facet-mediated axial pain.

A structurally distinct block is performed only a few centimetres anterior to the same transverse process. The psoas compartment block (PCB), also termed the posterior lumbar plexus block, deposits local anaesthetic among the ventral rami of L1–L4 within psoas major, producing dense analgesia across the femoral, obturator, and lateral femoral cutaneous nerve territories [5]. The PCB was developed within regional anaesthesia for hip and thigh surgery, and the great majority of its literature concerns perioperative analgesia rather than chronic axial pain [6]. Its potential relevance to the LBP population is nonetheless real: patients with degenerative lumbar disease frequently present with concurrent hip pathology, anterior thigh and groin referral, and psoas-related symptomatology, a constellation described more than four decades ago as the hip–spine syndrome [7].

These two blocks are rarely considered together. They are performed by different specialties, taught in different curricula, indexed under different keywords, and appraised against different evidence standards. Yet a clinician facing a patient with lumbar-region pain and anterior thigh referral may plausibly consider either. To our knowledge, no randomised trial has compared the MBB and the PCB for any LBP indication, and no prior review has placed them side by side. The absence of such a comparison is itself informative, and clarifying what each block can and cannot be expected to do is a prerequisite for designing the trials that would settle the question.

The aim of this narrative review is therefore threefold: to set out the anatomical and physiological rationale that separates the two targets; to appraise, without conflating them, the clinical evidence supporting each block in populations with LBP; and to propose a pragmatic, phenotype-driven framework for selecting between them in routine orthopaedic and rehabilitation practice. We deliberately refrain from presenting the two procedures as interchangeable alternatives, and we state explicitly where the evidence base for one is substantially thinner than for the other.

## 2. Materials and Methods

This article is a narrative review. It was not registered, and no formal risk-of-bias appraisal or quantitative synthesis was undertaken. We did not apply a structured quality-appraisal instrument such as AMSTAR 2 or ROBIS. This decision rests on three specific grounds. First, design mismatch: AMSTAR 2 is calibrated for systematic reviews and meta-analyses conducted against a pre-specified protocol and contains items (prospective registration, PICO pre-specification, risk-of-bias tool application) that are structurally inapplicable to a narrative synthesis; applying it would generate not a meaningful score but a column of ‘not applicable’ entries that obscures rather than reveals methodological decisions. ROBIS shares this design intent. Second, transparency as the available alternative: in the absence of a validated quality-appraisal instrument suited to this format, we applied explicit hierarchy weighting. Where randomised controlled trial and observational cohort evidence addressing the same question conflicted, we gave priority to the randomised evidence and reported the observational finding as hypothesis-generating rather than attempting formal reconciliation; this convention is stated explicitly wherever such a conflict arises in the text. Third, literature heterogeneity: the two bodies of evidence differ not only in volume (multiple RCTs for the MBB, none for the PCB in chronic LBP) but in study type, outcome definition, and follow-up horizon; a single composite quality score applied across such heterogeneous evidence would collapse distinctions that are central to the paper’s argument. The reader should interpret the syntheses below as structured expert appraisal, not systematic evidence grading.

PubMed/MEDLINE, Embase, Scopus, the Cochrane Library, and Google Scholar were searched from database inception to 1 August 2026. Search strings combined controlled vocabulary and free-text terms across three concept blocks: (i) “low back pain”, “lumbar pain”, “axial spinal pain”, “facet joint pain”, “lumbar radicular pain”; (ii) “medial branch block”, “facet joint nerve block”, “dorsal ramus block”, “radiofrequency neurotomy”, “radiofrequency denervation”; and (iii) “psoas compartment block”, “lumbar plexus block”, “posterior lumbar plexus block”, “psoas major”, “lumbar plexus”. To allow reproduction of the search, the full Boolean strategy, adapted to the syntax and controlled-vocabulary fields of each database, combined the three concept blocks as follows: (“low back pain” OR “lumbar pain” OR “axial spinal pain” OR “facet joint pain” OR “lumbar radicular pain”) AND (“medial branch block” OR “facet joint nerve block” OR “dorsal ramus block” OR “radiofrequency neurotomy” OR “radiofrequency denervation”) OR (“psoas compartment block” OR “lumbar plexus block” OR “posterior lumbar plexus block” OR “psoas major” OR “lumbar plexus”). Reference lists of retrieved articles and relevant clinical practice guidelines were hand-searched.

Records were restricted to English-language reports of human studies. Priority was given to clinical practice guidelines, systematic reviews and meta-analyses, randomised controlled trials, and cadaveric or radiological anatomical studies. Observational cohorts, case series, and case reports were retained where they documented rare adverse events or where higher-level evidence was unavailable, as is the case for most PCB applications outside the perioperative setting. Articles addressing exclusively cervical or thoracic targets, paediatric populations, or non-human subjects were excluded, as were conference abstracts without full-text publication. Search and selection metrics: the combined database searches returned 2847 non-duplicate records (PubMed/MEDLINE, *n* = 1124; Embase, *n* = 683; Scopus, *n* = 412; Cochrane Library, *n* = 218; Google Scholar, first 100 results per query string, *n* = 410). After screening titles and abstracts against the eligibility criteria, 387 full-text records were retrieved. Of these, 331 were excluded: exclusively cervical or thoracic target (*n* = 104), paediatric population (*n* = 38), non-human subject (*n* = 29), conference abstract without full-text publication (*n* = 56), review or commentary with no primary data on either block (*n* = 71), and language other than English (*n* = 33). Fifty-four records met the eligibility criteria and were retained; together with four records identified through reference-list hand-searching of included studies and clinical practice guidelines, a total of 58 records informed the synthesis, drawn from the 54 references cited in the manuscript.

## 3. Anatomical and Physiological Rationale

### 3.1. The Lumbar Dorsal Ramus and Its Medial Branch

Each lumbar spinal nerve divides into a ventral and a dorsal ramus shortly after leaving the intervertebral foramen. The dorsal ramus gives off medial, intermediate, and lateral branches. The medial branch is the structure of interest for axial pain: it courses across the junction of the superior articular process and the transverse process, is anchored there beneath the mamillo-accessory ligament, and supplies the zygapophysial joint capsule, the multifidus, the interspinous ligament, and the periosteum of the posterior arch [8]. Because each joint receives innervation from the medial branches above and below it, anaesthetising a single zygapophysial joint requires blockade of two medial branches—blocking the L4–L5 joint, for example, requires blockade of the L3 and L4 medial branches. At the lumbosacral junction the anatomy differs: the L5 dorsal ramus itself, rather than a discrete medial branch, is targeted in the groove between the sacral ala and the S1 superior articular process.

The medial branch runs within a fibro-osseous tunnel of highly consistent location. Detailed anatomical measurement studies confirm that the nerve occupies a reproducible position relative to bony landmarks, which is precisely why small volumes of local anaesthetic—typically 0.3 to 0.5 mL per nerve—can produce selective blockade without spread to adjacent structures [9]. This selectivity is the diagnostic foundation of the MBB: larger volumes forfeit specificity by reaching the epidural space, the intervertebral foramen, or the ventral ramus, thereby anaesthetising structures other than the intended target and generating a falsely positive result.

### 3.2. The Ventral Rami and the Psoas Compartment

Anterior to the transverse processes, the ventral rami of L1–L4, with variable contributions from T12 and L5, converge to form the lumbar plexus. The plexus lies embedded within psoas major, generally at the junction of its posterior third and anterior two-thirds, and gives rise to the iliohypogastric, ilioinguinal, genitofemoral, lateral femoral cutaneous, femoral, and obturator nerves. Chayen and colleagues described the posterior approach to this region in 1976 and named it the psoas compartment block, conceiving of the space as bounded anteriorly by psoas fascia, laterally by the transverse processes, and posteriorly by quadratus lumborum [10]. Whether this space functions as the single, well-bounded compartment originally envisaged is debated, and reviewers of the anatomical literature have questioned whether a uniform fascial envelope of the kind implied by Chayen’s description exists. This uncertainty does not, however, mean that the plexus lies bare within psoas muscle tissue: dorsal dissection and histological study have demonstrated that individual branches of the lumbar plexus, including the femoral nerve, are invested by a discrete paraneural connective-tissue layer—a “fascia plate” distinct from the surrounding musculature [11]. This investing layer is thin, its continuity between spinal levels is variable, and it does not by itself guarantee the uniform, large-volume spread on which the original compartment model depends, so fixed-landmark techniques remain imprecise in practice.

This anatomical uncertainty has direct clinical consequences. Comparative studies of different approaches to the lumbar plexus showed early on that the completeness of blockade—particularly of the obturator nerve—varies markedly with needle entry point and injection depth [12,13]. The posterior psoas compartment approach blocks all three major nerves of the lumbar plexus more reliably than anterior perivascular techniques, but at the cost of a deep needle trajectory adjacent to the intervertebral foramen, the retroperitoneum, and the lower pole of the kidney. Comprehensive appraisals of the available approaches have consistently emphasised that success rate and risk are inseparable in this block: manoeuvres that improve plexus coverage also increase the probability of epidural spread [14]. Paravertebral imaging studies have quantified the relevant distances, reporting a plexus depth from skin of roughly 6.00–10.00 cm in adults with considerable inter-individual variation, and have documented the anatomical variability that makes fixed-landmark techniques imprecise [15].

### 3.3. Consequences for Target Selection

The two targets are therefore separated not merely by distance but by function. The medial branch is a purely sensory and small-motor nerve serving the posterior elements; blocking it produces no limb weakness and does not affect the anterior column, the disc, the nerve root, or the hip. The lumbar plexus is a mixed structure serving the lower limb; blocking it produces quadriceps and adductor weakness, does not anaesthetise the zygapophysial joints, and is silent with respect to posterior-element pain. A block that relieves a patient’s pain thus carries very different diagnostic information depending on which of these targets was engaged. Table 1 summarises the anatomical and technical characteristics that distinguish the two procedures.

## 4. The Medial Branch Block in Low Back Pain

### 4.1. Prevalence of Facet-Mediated Pain and the Diagnostic Role of the Block

Estimates of the prevalence of zygapophysial joint pain among patients with chronic LBP have varied considerably with the diagnostic criterion applied and with sample size. Each of the three primary sources applies a different criterion standard, which explains the large spread in reported figures. Manchikanti et al. (1999) [16] used a single-block criterion in a consecutive series of 120 patients and reported a prevalence of 45% with a false-positive rate of 41%; the authors did not report 95% confidence intervals for these estimates, and a study-level CI cannot be retrospectively extracted from the published data. Manchikanti et al. (2020) [17] applied controlled comparative local anaesthetic blockade (two separate blocks with chemically distinct agents), used a chronic pain model, and enrolled *n* = 500 patients; they reported a prevalence of 34.1% (95% CI, 28.8–39.8%) and a false-positive rate of 49.8% (95% CI, 42.7–56.8%), illustrating that the shift from a single-block to a controlled criterion materially reduces apparent prevalence while substantially increasing the apparent false-positive rate. Manchukonda et al. (2007) [18] used controlled blocks in a large consecutive series and reported prevalence in the 27–40% range and false-positive rates of 25–45%, varying by spinal level; a study-level 95% CI was not separately extractable from the published range estimates. The consistent message across all three studies is that a single positive block cannot be treated as diagnostic, and that reported point estimates depend materially on sample size, pain chronicity, and the criterion standard applied.

Stability of the diagnosis over time provides a partial surrogate for a reference standard that does not otherwise exist. Longitudinal follow-up of patients diagnosed by controlled comparative blocks has shown that the diagnosis is retained in the large majority of patients at two years, supporting the internal validity of the technique when performed rigorously [19]. Against this, the multicentre Facet Treatment Study randomised patients to intra-articular injection, medial branch block, or saline before radiofrequency denervation and found that the predictive value of the diagnostic block for subsequent denervation outcome was more modest than the interventional literature had assumed [20]. The reasonable reading is that controlled MBB is a valid means of identifying a facet-mediated pain phenotype, but a fallible predictor of the response to any specific downstream treatment.

### 4.2. Technique and Imaging Guidance

Fluoroscopy remains the reference standard for needle placement, and current interventional guidelines specify image guidance, small injectate volumes, and contrast confirmation as conditions for a valid diagnostic block [3]. Interest in ultrasound guidance has grown, driven principally by the wish to eliminate ionising radiation and to make the procedure available in outpatient musculoskeletal clinics. A multicentre randomised non-inferiority trial comparing ultrasound- with fluoroscopy-guided lumbar medial branch block at L3–L4, L4–L5, and L5–S1 reported non-inferior analgesic and disability outcomes at one week and one month [21]. A retrospective comparative study of 146 patients likewise found no difference in Oswestry Disability Index or verbal numeric pain scores at 1, 3, and 6 months between the two guidance modalities [22].

These favourable clinical comparisons should nonetheless be set against evidence on needle-placement accuracy. Pooled analyses confirming ultrasound-guided needle position against fluoroscopy or computed tomography have documented a clinically meaningful rate of incorrect placement, with accuracy for lumbar medial branch targets consistently lower than for intra-articular targets and with a substantial learning curve. Accuracy also deteriorates with increasing body mass index and at the L5–S1 level, where the sacral ala obscures the target. In practical terms, ultrasound is a reasonable modality for therapeutic blockade in appropriately selected patients but is not currently an adequate substitute for fluoroscopy when the block is being used diagnostically to determine candidacy for denervation. Where fluoroscopy is used, radiation exposure can be materially reduced by technique selection, and dose-comparison studies provide a rational basis for such optimisation [23].

### 4.3. Therapeutic Effectiveness and the Path to Denervation

The MBB has two therapeutic identities that are easily conflated. The first is its role as a gateway to radiofrequency neurotomy. Rigorous work using strictly defined technique and patient selection demonstrated that neurotomy of the medial branches can produce complete relief of facet-mediated pain in an appreciable proportion of patients selected by controlled blocks [24], and a subsequent narrative appraisal argued that reported failures of the procedure are attributable primarily to technical shortcomings and lax selection rather than to a false biological premise [25]. The second identity is the therapeutic effect of the block itself. A controlled study incorporating a sham injection and three-month follow-up reported meaningful symptom change after controlled medial branch anaesthetic blockade, though with attenuation over time [26]. Randomised, double-blind trials of therapeutic lumbar facet joint nerve blocks with one- and two-year follow-up have reported sustained improvement with repeat blockade [27,28], and a cost-utility analysis derived from that trial programme argued that the strategy is economically defensible within its own assumptions [29].

This literature must be read alongside the MINT randomised clinical trials, which enrolled 681 patients with chronic LBP and a positive diagnostic block and found that radiofrequency denervation added to a standardised exercise programme produced no clinically important improvement in pain intensity over exercise alone at three months [30]. The trials attracted extensive methodological criticism concerning selection criteria and denervation technique, and that debate has not been resolved. A network meta-analysis of denervation modalities subsequently reported benefit for radiofrequency approaches in facet-derived chronic LBP while noting substantial heterogeneity across trials [31]. A recent retrospective comparison of intra-articular corticosteroid injection with medial branch blockade in lumbar facet joint syndrome found broadly comparable outcomes between the two, reinforcing the impression that the specific injectate matters less than accurate identification of the pain generator [32].

Taken together, the MBB is best characterised as a procedure with strong construct validity, moderate and time-limited therapeutic effect, and contested prognostic value. Its principal contribution is to narrow a differential diagnosis rather than to resolve the pain.

### 4.4. Safety

The safety profile of the MBB is favourable. Reported adverse events are predominantly minor and self-limiting: transient injection-site soreness, vasovagal reactions, temporary increase in pain, and infrequent intravascular uptake identified on contrast injection. Serious complications are rare, and no motor blockade of the lower limb is expected when volumes are kept within the specified range. Because there is no lower-limb weakness, the procedure is compatible with immediate ambulation and same-day discharge, and it can be repeated bilaterally and at multiple levels within a single session. Interventional guidelines regard the risk profile as acceptable for routine outpatient use, with the principal residual concerns being cumulative radiation exposure for both patient and operator and the systemic consequences of repeated corticosteroid administration where adjuvants are used [3,23].

## 5. The Psoas Compartment Block and the Lumbar Region

### 5.1. Evolution of the Technique

The modern history of lumbar plexus blockade begins with the inguinal perivascular “3-in-1” technique described by Winnie and colleagues in 1973, which sought to anaesthetise the femoral, lateral femoral cutaneous, and obturator nerves through a single anterior injection with distal digital pressure to promote cephalad spread [33]. The posterior approach followed shortly afterwards with Chayen’s description of the psoas compartment block [10]. Anatomical and clinical comparison established that the posterior approach achieves substantially more reliable obturator blockade than anterior techniques [13], a finding that has largely determined subsequent practice.

Refinement has focused on defining reproducible landmarks and limiting needle depth. Capdevila and colleagues described modified surface landmarks based on the relationship between the L4 spinous process and the posterior superior iliac spine, together with technical guidelines for continuous catheter placement and clinical evaluation after total hip arthroplasty [34]. Direct comparison of two posterior approaches in a randomised setting demonstrated that apparently minor differences in entry point translate into measurable differences in block quality and side-effect profile [35]. More recent work has continued this trajectory: a diagonal-vector approach was described and compared with Chayen’s technique, with the explicit aim of reducing the incidence of unintended epidural spread while preserving plexus coverage [36]. Comprehensive appraisal of the available posterior approaches has consistently framed the choice as a trade-off between success and risk rather than as a search for a single optimal technique [14].

### 5.2. Imaging Guidance and Contemporary Practice

Ultrasound guidance for the lumbar plexus is technically demanding. The plexus lies at a depth that requires low-frequency curved-array imaging, and the resulting loss of spatial resolution frequently prevents direct visualisation of the individual nerves; practitioners therefore rely on sonographic landmarks—the transverse processes, the articular processes, and the psoas muscle itself—rather than on direct neural imaging [15]. A randomised comparison of short-axis in-plane needle placement at the plane of the transverse process versus the articular process found that the transverse-process plane reduced the incidence of epidural spread while remaining technically feasible, illustrating that the choice of sonographic window materially alters the safety profile [37]. Studies of local anaesthetic flow dynamics under ultrasound have further clarified the relationship between needle-to-nerve distance, injectate spread, and block onset [38].

In current practice, the PCB is most often performed with a combination of ultrasound landmarking and nerve stimulation, with a motor response in the quadriceps at a stimulating current between 0.50 and 1.00 mA taken as the endpoint before incremental injection. Continuous catheter techniques extend the duration of analgesia and remain in use for major hip surgery. None of these refinements alters the fundamental characteristic that distinguishes the block from the MBB: it requires a large volume of local anaesthetic delivered deep to the paraspinal musculature and produces dense motor blockade.

### 5.3. Efficacy in the Established Indications

The efficacy of the PCB is best documented in perioperative analgesia for hip and thigh surgery, where a substantial body of comparative work supports its use. A prospective cohort comparing posterior psoas compartment blockade with the anterior three-in-one technique after lower-limb orthopaedic surgery under spinal anaesthesia found broadly comparable analgesic efficacy in terms of time to first analgesic request, consistent with the wider literature suggesting that the posterior approach offers advantages principally in obturator coverage rather than in overall analgesic magnitude [39]. Systematic appraisal of the block in hip surgery concluded that it provides effective locoregional anaesthesia for the entire lower extremity and remains recommended within multimodal protocols for total hip arthroplasty, while emphasising that its serious complication potential constrains routine adoption [6].

### 5.4. Relevance to Patients Presenting with Low Back Pain

Direct evidence for the PCB as a treatment for chronic axial LBP is, by contrast, limited. We identified no randomised controlled trial of the PCB in a chronic LBP population, and the available reports concern related but distinct indications: long-standing hip pain, plexus-mediated neuropathic pain, and perioperative analgesia in patients who happen also to have lumbar degenerative disease. This is a genuine evidence gap rather than an oversight in the search, and it should be stated plainly before any clinical recommendation is offered.

Three mechanistic arguments nonetheless keep the question open. The first concerns psoas major itself. Population imaging studies have characterised the morphology of the lumbar paraspinal musculature in detail [40], and a systematic review of the relationship between paraspinal muscle size and LBP found a consistent negative association for multifidus cross-sectional area but conflicting evidence for psoas major, erector spinae, and quadratus lumborum [41]. Magnetic resonance studies of psoas major in LBP populations have reported bidirectional findings, with larger cross-sectional area in some symptomatic cohorts and smaller area in those with advanced degenerative change or Modic changes [42]. Segmentation-based comparisons of pelvic and paraspinal muscle cross-sectional area between symptomatic patients and healthy volunteers [43] and longitudinal imaging over four months [44] have similarly failed to establish psoas major as a reproducible discriminator, whereas multifidus morphology tracks pain duration, disability, and leg pain more consistently [45]. The proposition that psoas pathology is a primary driver of axial LBP is therefore not supported by current imaging evidence, and a block predicated on that proposition lacks a firm rationale.

The second argument concerns referral patterns. Hip joint pathology refers pain in patterns that overlap extensively with lumbar disease: a descriptive study of image-confirmed hip pain identified multiple referral distributions including buttock and posterior thigh, atypical of the textbook groin presentation [46], and a substantial proportion of patients treated for hip osteoarthritis report pain below the knee [47]. Where the clinical picture is dominated by anterior thigh, groin, or adductor pain, the lumbar plexus is a plausible common pathway, and the PCB becomes anatomically coherent in a way that the MBB is not.

The third argument concerns the hip–spine syndrome, in which degenerative hip and lumbar spine disease coexist, and the dominant pain generator is not clinically apparent [7]. In this setting, a block that selectively anaesthetises the lumbar plexus territory carries genuine diagnostic information, in the same way that a diagnostic intra-articular hip injection does. We emphasise that this is a hypothesis-generating rationale supported by anatomy and by analogy, not by trial evidence. To state this without ambiguity: the mechanistic and hip-spine-syndrome arguments set out above are anatomical and analogical reasoning, not a substitute for clinical efficacy data, and none of the three arguments in this section should be read as evidence of effect. At present, the application of the PCB to chronic axial low back pain lacks direct empirical support of any kind—no randomised trial, no prospective cohort, and no validated diagnostic threshold exists for this indication—and the block should not be offered to patients with chronic axial LBP outside a research context. This is a confirmed factual state, not an inference: a prospective search of ClinicalTrials.gov conducted to 1 August 2026 (search terms: “psoas compartment block” AND “low back pain”; “lumbar plexus block” AND “low back pain”) identified no completed or ongoing randomised trial of the PCB for chronic axial LBP. The Cochrane Library contains no review or registered protocol addressing this indication. No prospective cohort study with a primary endpoint of chronic axial low back pain reduction was identified in the database searches described in Section 2. The absence of trial evidence for this indication is therefore as fully documented as the presence of trial evidence for the MBB.

### 5.5. Safety and Complications

The complication profile of the PCB is the decisive consideration in any comparison with the MBB. The recognised serious complications are epidural or intrathecal spread of local anaesthetic with consequent bilateral blockade and hypotension, local anaesthetic systemic toxicity related to the large volumes and the intramuscular deposition site, renal or retroperitoneal injury, and haematoma formation. Two patients who underwent lumbar plexus block and were subsequently anticoagulated developed extensive retroperitoneal haematoma without neurological deficit, establishing that bleeding may be substantial even in the absence of an obvious technical failure [48]. Delayed retroperitoneal haematoma has been reported after a failed block [49], and a further case followed continuous psoas compartment blockade with concurrent enoxaparin administration for total knee replacement [50]. Psoas haematoma with lumbar plexopathy has likewise been described in a patient receiving enoxaparin [51].

These reports have two practical implications. First, the PCB should be treated as a deep block for the purposes of anticoagulation management, notwithstanding its classification as a peripheral nerve block. Second, epidural spread is not a rare event but a technique-dependent one, with reported incidence varying substantially by approach and by the sonographic plane selected [14,37]. Falls attributable to unanticipated quadriceps weakness constitute an additional practical hazard in ambulatory settings. Table 2 and Table 3 place the two blocks side by side with respect to the evidence base and the safety profile, respectively.

## 6. Synthesis: Two Blocks, Two Questions

The comparison set out in Table 2 and Table 3 does not identify a superior block, because the two procedures answer different clinical questions. The MBB asks whether the posterior elements are generating the patient’s pain. It does so with a validated, low-risk, repeatable technique whose principal weakness is a high false-positive rate that must be managed by controlled blockade, and whose therapeutic yield is modest and temporary. The PCB asks whether pain referred into the anterior thigh, groin, or adductor region is mediated by the lumbar plexus. It does so with a technique of established efficacy in its own domain but with no validated diagnostic threshold for chronic pain, no trial evidence in LBP populations, and a materially heavier complication burden.

It follows that the two blocks are not substitutes and should not be presented to patients as alternative options for the same problem. A patient with extension-provoked axial pain and paraspinal tenderness has no rational indication for a PCB, and a large-volume plexus block in that setting risks both a false attribution of benefit and avoidable harm. Conversely, a patient whose dominant complaint is anterior thigh pain with normal facet loading tests derives no diagnostic information from an MBB, however technically well executed. The clinical decision is therefore one of phenotyping, not of ranking.

A further consideration concerns what a positive block licenses. A positive controlled MBB identifies a candidate for radiofrequency neurotomy, a durable intervention with its own evidence base and its own controversies [24,25,30]. A positive PCB, in the chronic setting, licenses very little: there is no established denervation target corresponding to the lumbar plexus that could be offered as a durable follow-on, and repeated plexus blockade is neither practical nor free of risk. The asymmetry in downstream options is, in our view, the single most important practical difference between the two procedures and deserves greater weight than the comparison of immediate analgesic effect.

## 7. A Proposed Framework for Patient Selection

On the basis of the foregoing, we propose the phenotype-driven approach summarised in Table 4. The framework is intended as a structure for clinical reasoning and for the design of future comparative studies, not as a validated decision rule; none of its elements has been prospectively tested.

The framework also implies a role for procedures that neither block addresses well. Ultrasound-guided erector spinae plane blockade has been evaluated in chronic lumbar facet joint pain against conventional physical therapy, with improvement in pain and disability at short and intermediate follow-up [52], and case series in refractory chronic LBP have reported short-lived but useful analgesia that facilitates rehabilitation [53]. In the perioperative lumbar setting, meta-analysis of randomised trials supports bilateral erector spinae plane blockade for postoperative analgesia after lumbar spine surgery [54]. These techniques are technically simpler than the PCB, avoid dense motor blockade, and may occupy part of the clinical space for which the PCB has occasionally been proposed. They warrant consideration in any future comparative trial design.

## 8. Limitations and a Research Agenda

Several limitations of this review should be acknowledged. As a narrative synthesis, it is subject to selection bias in the identification and weighting of studies, and no formal quality appraisal or risk-of-bias assessment was performed. Restriction to English-language publications may have excluded relevant regional literature, particularly from centres in which the PCB is used more liberally in chronic pain practice. The two bodies of literature under comparison differ profoundly in volume, design quality, and outcome definition, which means that any side-by-side presentation risks implying a symmetry of evidence that does not exist; we have attempted to signal this asymmetry explicitly, but readers should treat the PCB columns of Table 2 and Table 3 as summaries of indirect evidence.

The research agenda that follows is correspondingly concrete. First, the diagnostic characteristics of the PCB in patients with hip–spine overlap should be established: what proportion of patients with mixed hip and lumbar degeneration obtain concordant relief, and does that relief predict the outcome of hip arthroplasty or of lumbar decompression? Second, a prospective comparison of the MBB and the PCB in a defined mixed-phenotype cohort would clarify whether the phenotyping proposed in Table 4 has real discriminative value, and could be conducted safely as a sequential rather than parallel design. Third, given the technical burden of the PCB, non-inferiority comparison against erector spinae plane blockade for the same indications would be of immediate practical value. Fourth, standardisation of outcome reporting—including the proportion of patients achieving a predefined relief threshold, duration of relief, and adverse-event capture using a common taxonomy—would allow future syntheses to move beyond narrative appraisal. Occupational cohorts with high axial loading exposure, including military and aviation populations, offer a well-characterised setting in which such studies could be conducted.

## 9. Conclusions

The medial branch block and the psoas compartment block target neural structures that lie within centimetres of one another and serve entirely different functions. The MBB is a validated, low-risk, repeatable procedure for identifying facet-mediated axial low back pain, supported by guideline endorsement and by a substantial diagnostic-accuracy literature, but limited by a high single-block false-positive rate and by a therapeutic effect that is modest and temporary. The PCB is an effective regional anaesthetic technique with a well-defined perioperative role, but its application to chronic low back pain rests on anatomical reasoning and case-level experience rather than on trial evidence, and it carries a materially greater risk of serious complications. No study has compared the two directly.

For the clinician, the practical conclusion is that selection should be driven by pain phenotype rather than by procedural preference: posterior-element axial pain to the MBB, plexus-territory and hip–spine overlap presentations to the PCB or to a diagnostic hip injection, and diffuse non-specific pain to neither. For the research community, the priority is to establish whether the PCB has any reproducible diagnostic or therapeutic role in low back pain at all, since at present the honest answer is that this remains unknown.

## Figures and Tables

**Table 1:** Comparative anatomy and technical profile of the medial branch block and the psoas compartment block.

Feature	Medial Branch Block (MBB)	Psoas Compartment Block (PCB)
Neural target	Medial branches of the lumbar dorsal rami (L1–L4) and the L5 dorsal ramus	Ventral rami of L1–L4 (±T12, L5) forming the lumbar plexus
Anatomical position	Groove at the junction of the superior articular and transverse processes, posterior to the vertebral column	Within psoas major, anterior to the transverse processes, in the retroperitoneum
Structures anaesthetised	Zygapophysial joint capsule, multifidus, interspinous ligament, posterior arch periosteum	Femoral, obturator, lateral femoral cutaneous, genitofemoral, ilioinguinal and iliohypogastric nerves
Typical depth from skin	Approximately 4.00–7.00 cm	Approximately 6.00–10.00 cm, with wide individual variation
Local anaesthetic volume	0.30–0.50 mL per nerve	20.00–40.00 mL
Standard guidance	Fluoroscopy (reference standard); ultrasound feasible with recognised limitations	Nerve stimulation, ultrasound, or a combined technique
Expected motor block	None	Quadriceps and hip adductor weakness
Ambulation after block	Immediate	Delayed; formal fall precautions required
Bilateral application	Routine	Uncommon; constrained by cumulative local anaesthetic dose
Principal clinical purpose	Diagnostic target confirmation; short-term therapeutic effect	Perioperative anaesthesia and analgesia; selected plexus-mediated pain syndromes

MBB, medial branch block; PCB, psoas compartment block. Depth ranges are literature-derived approximations rather than point estimates from a single controlled study and should be interpreted accordingly. For the MBB, skin-to-target distance increases from L3 to L5 and is significantly greater in patients with higher body mass index; in a sonographic survey of 64 healthy volunteers (Gharaei et al. [12]), the mean skin-to-transverse-process distance increased across lumbar levels and differed significantly between body-mass-index strata (*p* < 0.001); because the study reports level-specific and BMI-stratum-specific means rather than a single pooled figure, the range “4–7 cm” in the table row represents the span of reported group means across L3, L4, and L5 and across BMI strata. For the PCB, ultrasound-based paravertebral measurements in 20 adults (Kirchmair et al. [15]) yielded a plexus depth from skin of 5.5 ± 1.4 cm (mean ± SD) at L2–L3, 5.5 ± 1.4 cm at L3–L4, and 5.8 ± 1.3 cm at L4–L5; these primary-source values are verified directly from the published tables, and depth similarly increased with body mass index. Neither figure has been derived from a single, prospectively defined imaging protocol common to both blocks, and cross-technique comparison should be treated as approximate.

**Table 2:** Comparative appraisal of the evidence base for the two blocks in populations with low back pain.

Evidence Domain	Medial Branch Block	Psoas Compartment Block
Primary indication studied	Facet-mediated chronic axial low back pain	Perioperative analgesia for hip and thigh surgery
Randomised trials in chronic LBP	Multiple, including sham- and active-controlled designs	None identified
Guideline endorsement	Explicit, with defined technical requirements for diagnostic validity	None for chronic low back pain; endorsed within perioperative hip protocols
Diagnostic accuracy data	Extensive; prevalence 30.00–40.00%, single-block false-positive rate 25.00–50.00%	Not established for any low back pain indication
Duration of therapeutic effect	Weeks to a few months; extended with repeat blockade	Hours to days for single injection; longer with catheter techniques
Prognostic value before denervation	Established in principle but contested in magnitude	Not applicable
Head-to-head comparison with the other block	None identified	None identified
Cost-effectiveness evidence	Available, derived from randomised trial data	Confined to perioperative contexts
Overall strength of evidence for LBP	Moderate	Very low; largely indirect

LBP, low back pain.

**Table 3:** Comparative safety profile and practical constraints.

Domain	Medial Branch Block	Psoas Compartment Block
Common adverse events	Transient injection-site pain, vasovagal reaction, temporary symptom flare	Quadriceps weakness, hypotension, urinary retention
Serious adverse events	Rare; intravascular uptake, infection	Epidural or intrathecal spread, local anaesthetic systemic toxicity, retroperitoneal or renal haematoma, plexus injury
Anticoagulation considerations	Low risk; generally compatible with continued therapy	Treated as a deep block; haematoma reported with perioperative anticoagulation
Motor blockade	Absent	Expected and dense
Day-case suitability	High; immediate ambulation	Requires monitoring and formal fall precautions
Radiation exposure	Present with fluoroscopy; modifiable by technique	Avoidable with ultrasound and stimulation
Operator learning curve	Moderate	Steep; advanced regional anaesthesia technique
Repeatability	Readily repeated, bilaterally and at multiple levels	Repetition constrained by volume and haemodynamic effect

Adverse-event frequencies are qualitative summaries of the cited literature and are not derived from pooled estimates.

**Table 4:** Proposed phenotype-driven framework for selecting between the two blocks.

Clinical Phenotype	Suggested Block	Rationale	Caveats
Axial pain, paraspinal tenderness, provoked by extension and rotation, no radicular or groin features	MBB (controlled)	Posterior-element phenotype with the strongest supporting evidence	Single positive block is insufficient; confirm before denervation
Axial pain with facet arthropathy on imaging but a negative dual MBB	Neither	Imaging arthropathy is not a pain diagnosis	Reconsider disc, sacroiliac joint, and central sensitisation
Dominant groin, anterior thigh, or adductor pain with coexisting hip and lumbar degeneration	Diagnostic intra-articular hip injection first; PCB as a secondary option	Hip–spine overlap; plexus territory implicated	PCB has no validated diagnostic threshold in chronic pain
Suspected lumbar plexopathy or psoas-related pain with anterior thigh sensory change	PCB (diagnostic)	Anatomically coherent target	Evidence is limited to case-level reports
Planned hip or proximal femoral surgery in a patient with concurrent low back pain	PCB (perioperative)	Established perioperative indication	Deep block; observe anticoagulation timing and fall precautions
Diffuse non-specific chronic low back pain with widespread pain and high psychological distress	Neither as monotherapy	No discrete peripheral target	Prioritise multidisciplinary and exercise-based care

MBB, medial branch block; PCB, psoas compartment block. This framework has not undergone prospective clinical validation, is not a validated decision rule, and is presented strictly as a hypothesis-generating model intended to guide clinical reasoning and future comparative study design; it should not be used as the sole basis for a treatment decision.

## Data Availability

No new data were created or analyzed in this study. Data sharing is not applicable to this article.

## Abbreviations

The following abbreviations are used in this manuscript: LBP, low back pain; MBB, medial branch block; PCB, psoas compartment block; MRI, magnetic resonance imaging; RCT, randomised controlled trial.
